# Meta-analysis of the relationship between methylenetetrahydrofolate reductase C677T and A1298C polymorphism and venous thromboembolism in the Caucasian and Asian

**DOI:** 10.1042/BSR20200860

**Published:** 2020-07-10

**Authors:** Miao Gao, Na Feng, Meixia Zhang, Xinyu Ti, Xiuping Zuo

**Affiliations:** 1 The First Affiliated Hospital of Air Force Medical University (XiJing Hospital), Respiratory and Critical Care Medicine, Xi’an, Shaanxi 710032, P.R. China; 2 School of Life Science and Technology, Xi'an Jiaotong University, No.28, Xianning West Road, Xi’an, Shaanxi 710049, P.R. China; 3 The First Affiliated Hospital of Air Force Medical University (XiJing Hospital), Department of Nursing, Xi’an, Shaanxi 710032, P.R.China

**Keywords:** meta-analysis, Methylenetetrahydrofolate reductase, polymorphism, venous thromboembolism

## Abstract

Recent years, it is a highly debated topic that whether methylenetetrahydrofolate reductase (MTHFR) C677T polymorphism and A1298C polymorphism could increase susceptibility to venous thromboembolism (VTE) in the Asian and Caucasian. Therefore, we expect to settle that controversy evidentially. Basic methods: Electronic databases (Pubmed, embase, Cochrane library, scopus, OvidSP, Wiley Online library, Springer link, EBSCO, Elsevier Science Direct, Google scholar) without date limitation were searched. Crude odds ratio (OR) along with 95% confidence interval (95% CI) was calculated to assess the association quantitatively. Finally, a total of 37 eligible studies were included, containing 31 for MTHFR C677T polymorphism and 6 for MTHFR A1298C polymorphism. The pooled results suggested that MTHFR C677T mutation may increase susceptibility to VTE in reverse recessive model (CC+CT vs TT): OR = 0.68 (0.56, 0.83), reverse dominant model (CC vs CT +TT): OR = 0.82 (0.72, 0.94), heterozygote model (CT vs TT): OR = 0.65 (0.52, 0.81), homozygote model (CC vs TT): OR = 0.73 (0.60, 0.89) and allele model (C vs T): OR = 0.80 (0.71, 0.90). Subgroup analysis about Asian also support that results, but Caucasian group not. In addition, MTHFR A1298C polymorphism may be not related to VTE in all genetic model. The results of meta-analysis indicated that MTHFR C677T polymorphism might increase the risk of VTE, especially in Asian population.

## Introduction

Venous thromboembolism (VTE) is a common clinical vascular syndrome, consisting of deep vein thrombosis (DVT) and pulmonary embolism (PE), which are two different forms of the same disease [1] At present, venous thrombosis has become the third cause of cardiovascular disease and common complications of cancer, such as lung cancer [2]. VTE is a complex multi-factor disease, in which gene mutation plays an important role [3]. However, there are great ethnic and regional characteristics of gene mutation in VTE. Therefore, exploring the susceptible genes to provide the basis for the prevention and treatment of VTE will be one of the important research directions of comprehensive therapy for vascular diseases and cancer.

Methylenetetrahydrofolate reductase (MTHFR) is a homocysteine (Hey) metabolic regulatory enzyme. It could reduce N5, N10-methylene tetrahydro folic acid to N5- methyl tetrahydro folic acid, and the latter has the ability to maintain the stability of plasma Hey. The decrease of MTHFR activity will give rise to impaired Hey methylation and further hyperhomocysteinemia, which could destroy vascular endothelium and change platelet function as well as blood coagulation state, finally participating in the pathogenesis of VTE. Both the mutation of MTHFR gene at 677site from base cytosine (C) to thymine (T) and the mutation of MTHFR gene at 1298site from adenine (A) to cytosine (C) could cause amino acid mistranslation, further decrease MTHFR activity and increase Hey level.

In recent years, many studies about the relationship between MTHFR gene polymorphism and the risk of VTE have been reported, but with inconsistent conclusions. Some hold the view that MTHFR/C677T was a significant risk factor of VTE, which demonstrates the association of MTHFR C677T polymorphism with the susceptibility to VTE [4], but some not [5]. In addition to MTHFR C677T polymorphism, it is also a highly debated issue that whether MTHFR A1298C polymorphism could increase the susceptibility to VTE. Therefore, we conducted this meta-analysis to explore the correlation between MTHFR C677T polymorphism as well as MTHFR A1298C polymorphism and the risk of VTE, providing theoretical basis for the prevention and treatment of VTE.

## Materials and methods

### Search strategy and selection criteria

This systematic review and meta-analysis is reported in accordance with the Preferred Items for Systematic Reviews and Meta-analysis (PRISMA) Statement. Literature was retrieved by formal search of electronic databases (Pubmed, embase, Cochrane library, scopus, OvidSP, Wiley Online library, Springer link, EBSCO, Elsevier Science Direct, Google scholar) without date limitation. To achieve the maximum sensitivity of the search strategy, we used appropriated free text and thesaurus terms including “methylenetetrahydrofolate reductase or MTHFR”, “Venous thromboembolism or VTE”, “polymorphism or mutation or variant”. We also search reference lists of related articles by hand to obtain more studies. The retrieval strategy of Pubmed is as follows: (((((polymorphism[Title/Abstract] OR mutation[Title/Abstract] OR variant[Title/Abstract])) OR “Polymorphism, Genetic”[Mesh])) AND ((deep venous thrombosis[Title/Abstract]) OR “Venous thromboembolism”[Mesh])) AND (((Methylenetetrahydrofolate Reductase (NADPH) or Methylene-THF Reductase (NADPH) or Methylenetetrahydrofolate Reductase or 5,10-Methylenetetrahydrofolate Reductase (NADPH) or Methylene Tetrahydrofolate Reductase or Tetrahydrofolate Reductase, Methylene)) OR “Methylenetetrahydrofolate Reductase (NADPH2)”[Mesh]).

Inclusion criteria: (1) Patients with VTE, including venous thrombosis and deep venous thrombosis; (2) Methylenetetrahydrofolate reductase C677T polymorphism and A1298C polymorphism; (3) Sufficient genotype data; (4) *P* value for Hardy–Weinberg equilibrium test > 0.05; (5) Case–control design.

### Data extraction and quality assessment

Two authors independently extracted the original data. As recommended by the Cochrane Non-Randomized Studies Methods, Newcastle–Ottaw scale (NOS) was used to assess the quality of included researches and a total score of included studies ranging from 7 to 9 was deemed high quality. Disagreement was resolved by discussion. The extracted data were consisted of the follow items: the first author’s name, publication year, country, race, genotype distribution data, total number of cases and controls.

### Statistical analysis

Meta-analysis was performed to calculate pooled ORs (Odds ratios) with 95% CI (Confidence interval) by using Review manager 5.3. Heterogeneity among studies was assessed by *I*^2^ statistic. I^2^> 50% is indicative of heterogeneity [6], random effects model will be used. Otherwise, fixed effect will be implemented. Chi-square distribution was employed to measure the deviation of genotype distribution from Hardy–Weinberg equilibrium in control group. Subgroup analysis was conducted to explore the sources of heterogeneity and the differences between races. We also perform sensitive analysis by changing effect models. Finally, funnel plots were carried out to evaluate publication bias. The *P*-value <0.05 in all tests was considered significant.

## Results

### Flowchart and characteristic of including studies

**Figure 1:**
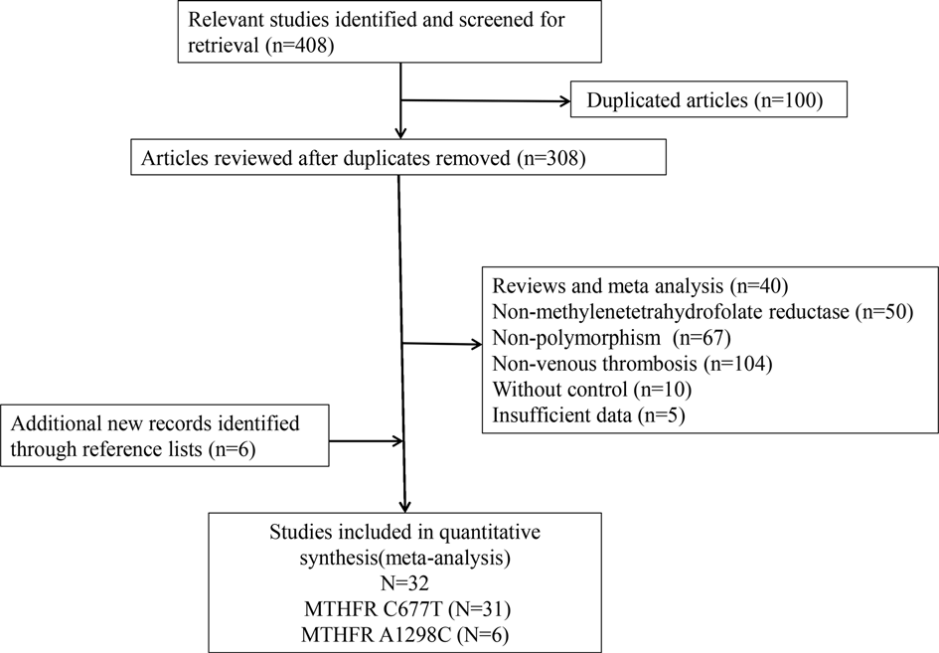
Flow diagram for literature selection

There are 32 eligible studies meeting to our inclusion criteria [4,5,7–36], including 31 papers for MTHFR C677T polymorphism [4,5,7–25,27–36] and 6 papers for MTHFR A1298C polymorphism [12,15,19,21,26,30]. The details of flow diagram for literature selection were shown in Figure 1. Among included studies, a total of 15 for the Asian [5,8,9,11,13,14,17,18,21–24,27,34,36], mainly in China, and 17 for the Caucasian [4,7,10,12,15,16,19,20,25,26,28–33,35]. Due to the comprehensive search, the publication year is from 1999 to 2019. The total sample size is nearly 20,000, containing 8223 patients and 10,859 controls. The main features of eligible studies are summarized in Tables 1 and 2.

**Table 1:** Characteristics of include studies about MTHFR C677T polymorphism

Author, year	Country	Race	Case group				Control group				pHWE	NOS
			CC	CT	TT	Total	CC	CT	TT	Total		
Jang, 2013	South Korea	Asian	74	82	47	203	140	203	60	403	0.62	8
Xu, 2019	China	Asian	42	28	31	101	70	26	24	120	0.10	8
Yin, 2012	China	Asian	171	157	112	440	182	190	68	440	0.30	7
Kailibinuer, 2012	China	Asian	22	31	35	88	30	45	11	86	0.65	7
Wang, 2004	China	Asian	13	28	17	58	19	32	7	58	0.51	7
Qiu, 2002	China	Asian	23	32	14	69	42	47	12	101	0.98	7
Hsu, 2001	China	Asian	60	40	7	107	55	44	8	107	0.98	8
Lu, 2002	China	Asian	18	42	30	90	31	66	46	143	0.73	8
Guo, 2002	China	Asian	4	26	33	63	16	35	29	80	0.66	7
Zheng, 2000	China	Asian	12	31	10	53	62	45	15	122	0.34	8
Lin, 2000	China	Asian	53	50	9	112	76	41	8	125	0.75	7
He, 2010	China	Asian	15	27	21	63	26	36	13	75	1.00	7
Li, 2015	China	Asian	71	107	68	246	97	155	40	292	0.21	8
Gao, 2008	China	Asian	16	34	14	64	14	39	11	64	0.21	7
Dong, 2013	China	Asian	16	37	15	68	15	41	12	68	0.23	8
Hsu TS, 2001	China	Asian	48	28	7	83	43	33	6	82	1.00	8
Karmadonova, 2014	Russia	Caucasian	76	79	19	174	226	201	34	461	0.50	7
Spiroski, 2008	Macedonia	Caucasian	20	33	10	63	34	35	11	80	0.92	7
Bezemer, 2007	Netherlands	Caucasian	2044	1891	440	4375	2245	2094	517	4856	0.68	8
Almawi, 2005	America	Caucasian	80	77	41	198	350	270	77	697	0.08	7
Miranda, 2002	Netherlands	Caucasian	67	90	14	171	233	186	42	461	0.86	6
Zalavras, 2002	Greece	Caucasian	70	82	24	176	117	153	30	300	0.14	7
Amparo, 2010	Spain	Caucasian	14	19	9	42	23	42	14	79	0.79	7
Tawfik, 2012	Egypt	Caucasian	20	4	25	49	22	1	1	24	0.01	7
Hanson, 2001	America	Caucasian	58	63	16	137	130	158	41	329	0.80	7
Ray, 2001	Canada	Caucasian	49	61	19	129	72	44	13	129	0.30	6
Gerald, 2000	Australia	Caucasian	67	73	15	155	122	141	35	298	0.84	6
Phillip, 2000	Canada	Caucasian	25	28	12	65	21	35	8	64	0.53	6
Ben, 2012	Tunisia	Caucasian	20	6	0	26	101	79	17	197	0.96	7
Kupeli, 2011	Turkey	Caucasian	49	24	7	80	78	26	0	104	0.35	8
Lupi-Herrera, 2018	Mexico	Caucasian	77	106	29	212	33	54	35	122	0.45	8

pHWE, *P* values for Hardy–Weinberg equilibrium test.

**Table 2:** Characteristics of include studies about MTHFR A1298C polymorphism

Author, year	Country	Race	Case group				Control group				pHWE	NOS
			AA	AC	CC	Total	AA	AC	CC	Total		
Hanson, 2001	America	Caucasian	60	62	15	137	164	139	26	329	0.90	8
Karmadonova, 2014	Russia	Caucasian	67	96	11	174	204	196	49	449	0.98	7
Martine, 1999	France	Caucasian	65	86	17	168	195	215	46	456	0.49	7
Li, 2015	China	Asian	163	65	18	246	180	97	15	292	0.92	7
Ray, 2001	Canada	Caucasian	68	49	12	129	69	49	11	129	0.86	8
Spiroski, 2008	Macedonia	Caucasian	32	29	2	63	38	39	3	80	0.18	8

pHWE, *P* values for Hardy–Weinberg equilibrium test.

## Meta-analysis results

### MTHFR C677T polymorphism and the susceptibility to Venous thromboembolism

**Figure 2:**
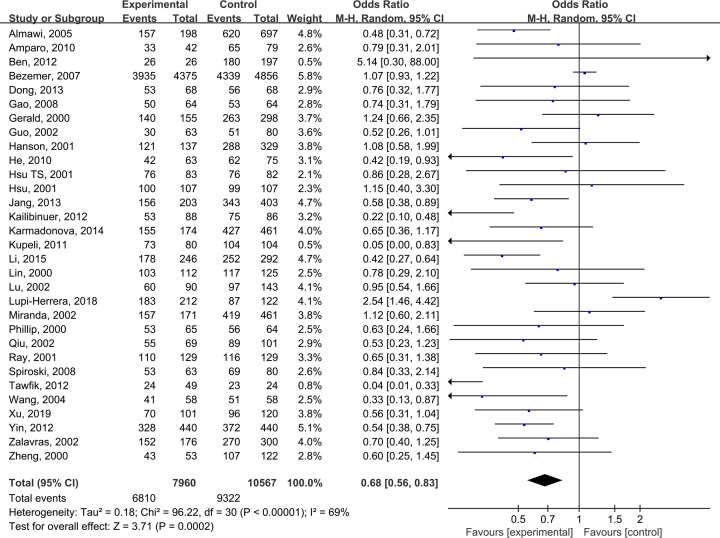
MTHFR C677T polymorphism and the susceptibility to Venous thromboembolism (CC+CT vs TT)

**Figure 3:**
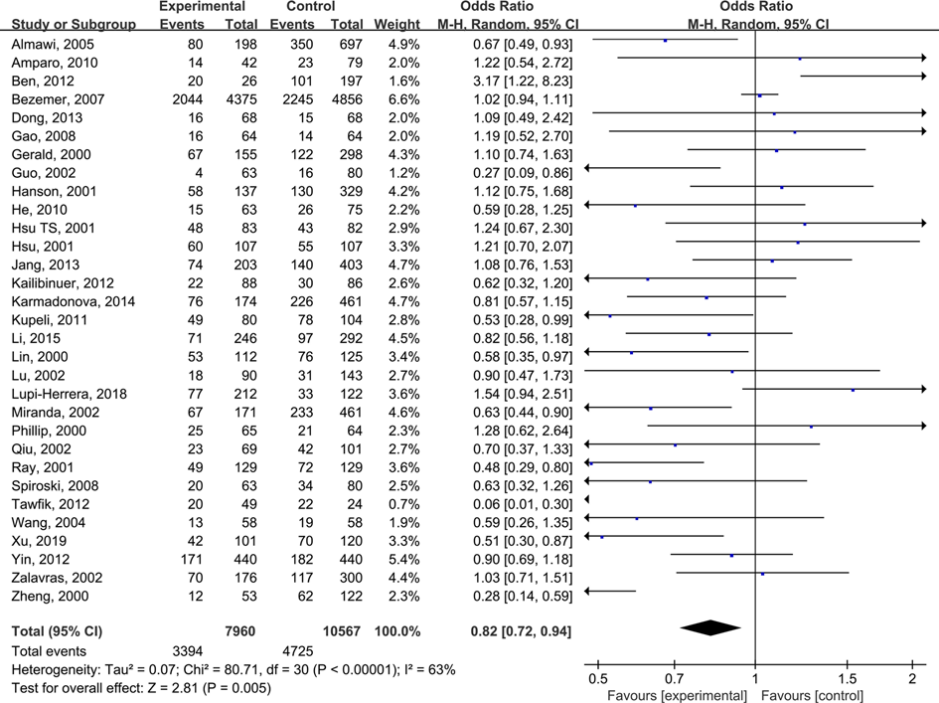
MTHFR C677T polymorphism and the susceptibility to Venous thromboembolism (CC vs CT+TT)

**Figure 4:**
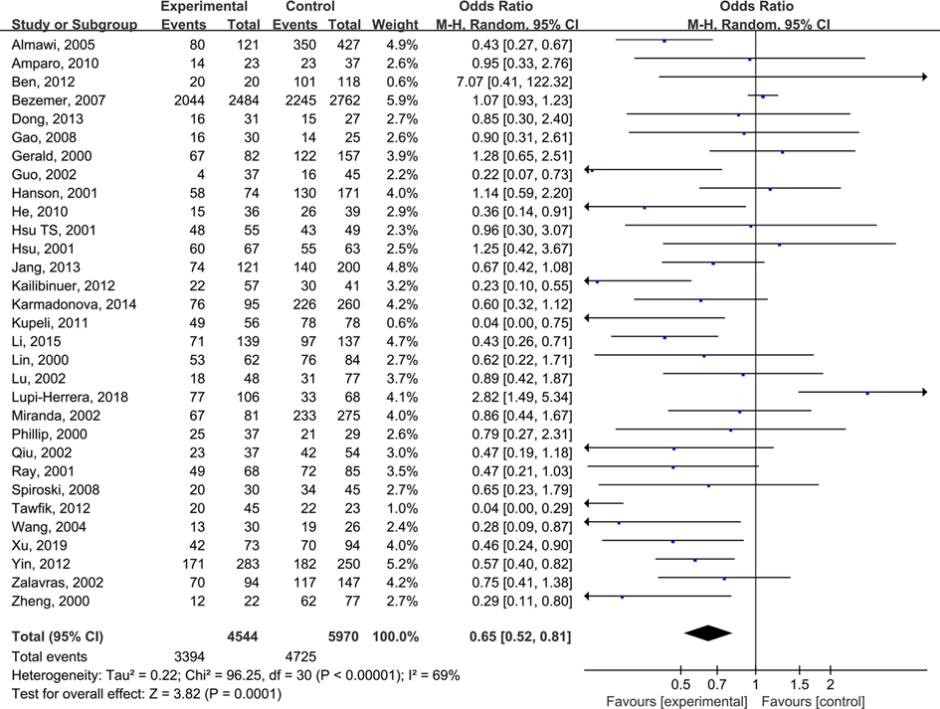
MTHFR C677T polymorphism and the susceptibility to Venous thromboembolism (CT vs TT)

**Figure 5:**
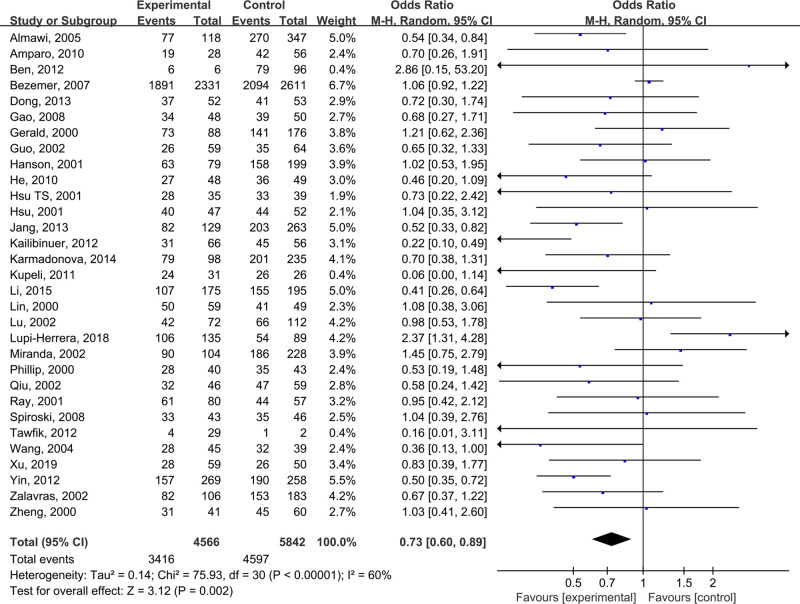
MTHFR C677T polymorphism and the susceptibility to Venous thromboembolism (CC vs TT)

**Figure 6:**
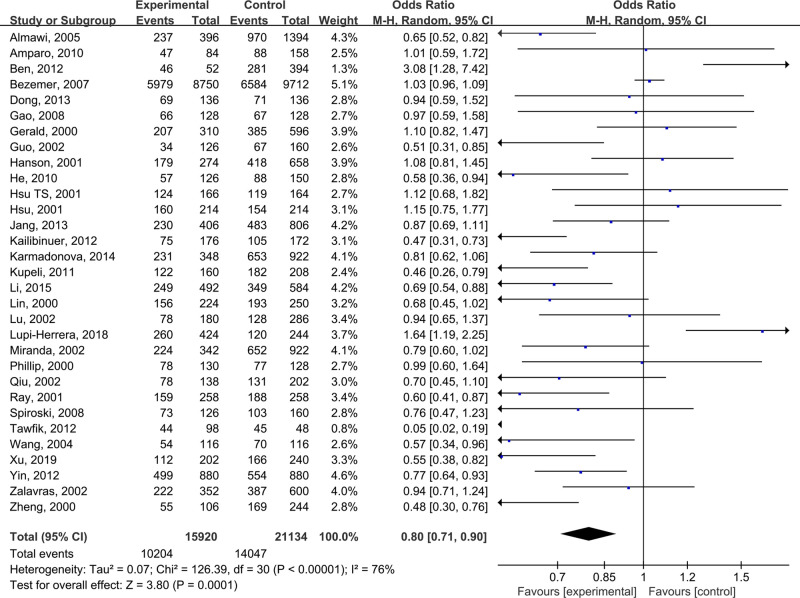
MTHFR C677T polymorphism and the susceptibility to Venous thromboembolism (C vs T)

The pooled results suggested that there were significant differences in all models of MTHFR C677T polymorphism, including CC+CT vs TT: OR = 0.68 (0.56, 0.83) (Figure 2), CC vs CT +TT: OR = 0.82 (0.72, 0.94) (Figure 3), CT vs TT: OR = 0.65 (0.52, 0.81) (Figure 4), CC vs TT: OR = 0.73 (0.60, 0.89) (Figure 5) and C vs T: OR = 0.80 (0.71, 0.90) (Figure 6). Due to significant heterogeneity, random effect models were used in all the comparisons. Subgroup analysis showed that, for the Asian, there was no heterogeneity in all the comparisons. But for the Caucasian, no significant association was observed, which tells us the source of heterogeneity and the difference between races (Table 3). Table 4 detailed the results of sensitive analysis, which demonstrated no significant change appeared in all pooled results after the transformation of random effect models into fixed effect models. The funnel plots showed good symmetry bias in all comparisons.

**Table 3:** Subgroup analysis of the relationship between MTHFR C677T polymorphism and the susceptibility to VTE

Comparison	Group	OR (95%CI)	*I^2^, P*
**CC+CT vs TT**	Total	0.68 (0.56, 0.83)	69%, <0.0001
	Asian	0.54 (0.46, 0.65)	10%, 0.34
	Caucasian	0.85 (0.63, 1.14)	69%, <0.0001
**CC vs CT+TT**	Total	0.82 (0.74, 0.93)	62%, <0.0001
	Asian	0.79 (0.66, 0.95)	38%, 0.06
	Caucasian	0.88 (0.72, 1.07)	71%, <0.0001
**CC vs TT**	Total	0.65 (0.52, 0.81)	69%, <0.0001
	Asian	0.52 (0.42, 0.65)	19%, 0.24
	Caucasian	0.81 (0.58, 1.12)	72%, <0.0001
**CT vs TT**	Total	0.73 (0.60, 0.89)	61%, <0.0001
	Asian	0.56 (0.46, 0.68)	13%,0.31
	Caucasian	0.93 (0.73, 1.19)	50%, 0.01
**C vs T**	Total	0.80 (0.71, 0.90)	76%, <0.0001
	Asian	0.74 (0.65, 0.83)	41%, 0.04
	Caucasian	0.88 (0.74, 1.04)	81%, <0.0001

OR, odds ratio.

**Table 4:** Sensitive analysis about MTHFR C677T and A1298C Polymorphism and VTE susceptibility

Comparison	Effect model	OR (95%CI)
**MTHFR C677T**		
CC+CT vs TT	Random	0.68 (0.56, 0.83)
	Fixed	0.80 (0.73, 0.87)
CC vs CT+TT	Random	0.82 (0.74, 0.93)
	Fixed	0.92 (0.87, 0.98)
CC vs TT	Random	0.65 (0.52, 0.81)
	Fixed	0.80 (0.73, 0.88)
CT vs TT	Random	0.73 (0.60, 0.89)
	Fixed	0.83 (0.75, 0.91)
C vs T	Random	0.80 (0.71, 0.90)
	Fixed	0.90 (0.87, 0.95)
**MTHFR A1298C**		
AA+AC vs CC	Random	0.97 (0.71, 1.32)
	Fixed	0.99 (0.74, 1.33)
AA vs AC+CC	Random	0.91 (0.77, 1.08)
	Fixed	0.91 (0.77, 1.08)
AA vs CC	Random	0.90 (0.66, 1.23)
	Fixed	0.91 (0.67, 1.25)
AC vs CC	Random	1.01 (0.67, 1.52)
	Fixed	1.05 (0.77, 1.43)
A vs C	Random	0.95 (0.83, 1.07)
	Fixed	0.95 (0.83, 1.07)

OR, odds ratio.

### MTHFR A1298C polymorphism and the susceptibility to VTE

**Figure 7:**
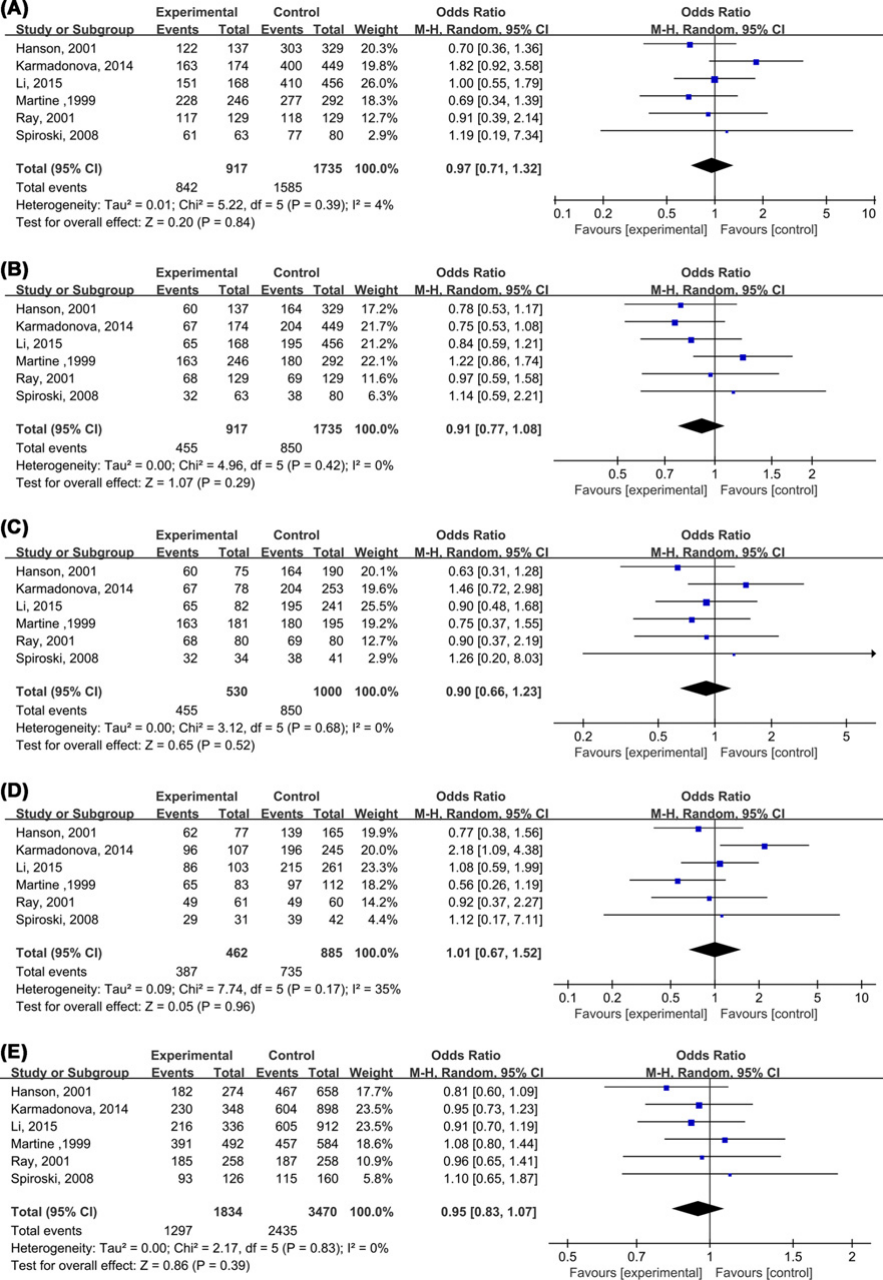
MTHFR A1298C polymorphism and the susceptibility to Venous thromboembolism ((**A**) AA+AC vs CC, (**B**) AA vs AC +CC, (**C**) AA vs CC, (**D**) AC vs CC, (**E**) A vs C)

Similar to C677T polymorphism, the comparisons of five models were conducted. As shown in Figure 7, none of any comparison exhibited significant difference statistically, with AA+AC vs CC: OR = 0.97 (0.71, 1.32) (Figure 7A), AA vs AC +CC: OR = 0.91 (0.77, 1.08) (Figure 7B), AA vs CC: OR = 0.90 (0.66, 1.23) (Figure 7C), AC vs CC: OR = 1.01 (0.67, 1.52) (Figure 7D) and A vs C: OR = 0.95 (0.83,1.07) (Figure 7E). Because of none heterogeneity, fixed effects were adapted. Sensitive analysis also suggested our results were stable (Table 4). Publication test failed to be conducted due to small sample included.

## Discussion

Although some meta-analysis about the relationship between the risk of VTE and MTHFR mutation have been reported, but the objects are mainly limited to C677T and Chinese. We not only expanded the population, including the Asian and Caucasian, but also explored the association of A1298C polymorphism and VTE susceptibility. Our results showed that, in all the comparisons of the gene phenotypic model, MTHFR C677T mutation could increase the risk of VTE in the Asian, but not in the Caucasian. In addition, there may be no association between MTHFR A1298C mutation and VTE susceptibility. Sensitive analysis and publication test suggested that our results were stable and reliable.

The human MTHFR gene, located on lp36.3 and with a cDNA length of 2.2 kb, is composed of 11(12) exons and 10(11) introns. MTHFR plays a key role in folic acid metabolism. The gene sequence of MTHFR is high conserved. If the gene sequence of 677 base cytosine C is mutated to thymine T, the valine generated by the mutation will replace the conserved alanine, which will lead to a serious decrease in the binding ability of MTHFR to flavin adenine dinucleotide [37]. The increased risk of many diseases caused by MTHFR mutation has been reported, such as congenital heart diseases [38], coronary artery disease [39], systemic lupus erythematosus [40] and cancer [41]. MTHFR’s thermal stability and enzyme activity were reduced due to the mutant T allele, resulting in hyperhomocysteine, which is an independent risk factor for VTE [42].

Zhang et al. [43] reported T allele, CT genotype, and TT genotype were associated with the risk of VTE in the Chinese population. Similar to our results, a pooled study of three Asian populations also showed the TT homozygous genotype could increase and the susceptibility to VTE [17]. Den et al. [44] reported that, in non-north American populations, the mutant T allele increased the risk of VTE compared with the wild-type C allele, but not in north American populations. The reason may be that higher intake of folic acid and riboflavin in north American populations reduces the risk of high homocysteine in carriers of the mutant T allele. Our study demonstrated that, regardless of gene models, C677T mutation couldn’t increase VTE susceptibility in the Caucasian.

1298 site of MTHFR is located in the exon 7 and encodes regulatory region of s-adenosine methionine. Likewise, the mutation of adenine (A) to cytosine (C) in this site causes glutamate to be replaced by alanine, decreasing the phosphorylation of serine and cysteine and thus affecting the expression of MTHFR as well [45]. As another MTHFR gene mutation, the relationship between MTHFR A1298C polymorphism and the risk of disease is also explored, such as Alzheimer’s disease [46] and lung cancer [47]. Our study is the first meta-analysis to explore the relationship between MTHFR A1298C polymorphism and VTE susceptibility. Six studies were included, in which only one paper was from the Asian, so we didn’t conduct subgroup analysis and publication test. Finally, no significant association was observed in any comparison of all gene models.

Because of the comprehensive search, large samples were included. Subgroup analysis suggested race is the source of heterogeneity and there exists great difference between the Asian and Caucasian. Of course, there were some limitation we need point out. Owe to insufficient data provided, confounding factors, including age, gender, body mass index, smoking status, drink abuse and other environmental factors, are difficult to fully be adjusted. Then, the controls were not uniformly defined, such as population- and hospital-based controls, and the latter may not necessarily be representative of the underlying source population.

In conclusions, our study uncovered that MTHFR C677T polymorphism may increase susceptibility to VTE in the Asian, but not in the Caucasian. There may be no association between MTHFR A1298C polymorphism and VTE. Our conclusion requires further focus on the effect of gene–gene and gene–environment interaction as well as different VTE types.

## Abbreviations

CI: confidence interval
MTHFR: methylenetetrahydrofolate reductase
NOS: Newcastle–Ottawa Scale
OR: odds ratio
pHWE: *P* values for Hardy–Weinberg equilibrium test
PRISMA: Preferred Items for Systematic Reviews and Meta-analysis
VTE: venous thromboembolism
